# First Example of a Heptazine-Porphyrin Dyad; Synthesis and Spectroscopic Properties

**DOI:** 10.3390/molecules27196698

**Published:** 2022-10-08

**Authors:** Frédérique Brégier, Vincent Sol, Yves Champavier, Laurent Galmiche, Clémence Allain, Pierre Audebert

**Affiliations:** 1 Laboratoire PEIRENE UR 22722, University of Limoges, 87000 Limoges, France; 2 BISCEm, NMR Platform, Centre de Biologie et de Recherche en Santé (CBRS), 87000 Limoges, France; 3 PPSM, Av. Des Sciences, 91100 Gif s. Yvette, CNRS UMR 8531, 61, Avenue du Président Wilson, CEDEX, 94235 Cachan, France; 4 XLIM, UMR CNRS 7252 123, Avenue Albert Thomas, CEDEX, 87060 Limoges, France

**Keywords:** Heptazines, Porphyrins, Dyad, Photochemistry, Electrochemistry

## Abstract

We have prepared the first example of a porphyrin linked to an heptazine photoactive antenna. The two entities, linked with an alkyl spacer, demonstrate the activity of both active moieties. While they behave electrochemically independantly, on the other hand the spectroscopy shows the existence of energy transfer between both partners.

## 1. Introduction

**Scheme 1:**
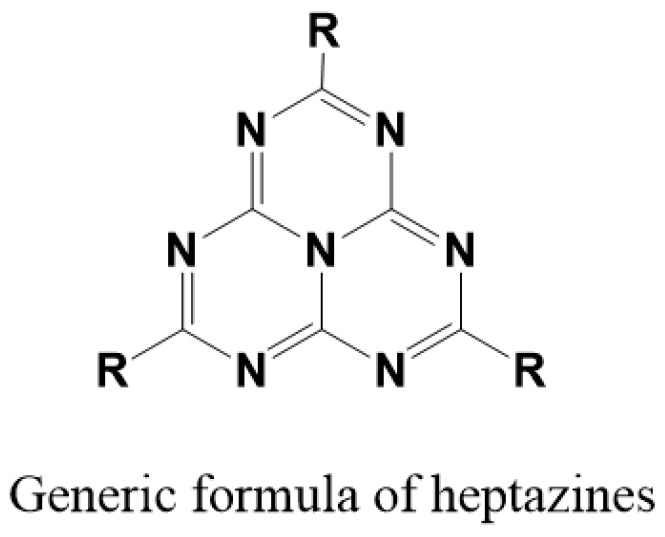
Generic formula of hetazines.

Porphyrins are ubiquitous heterocycles, whose implications and utilizations are countless, like biomedicine [1,2], sensors [3] or photovoltaics [4,5] (a specific journal is even dedicated exclusively to phtalocyanins and porphyrins. (“The Journal of Porphyrins and Phtalocyanins” a peer-referred review, World Scientific Ed., IF 1.8.)). However, their use, especially in catalysis applications, is usually related to their activation, which can be electrochemical [6,7], or photochemical [5,8,9] (to cite the most common ones). Among them, fluorinated porphyrins and derivatives [10] hold a special place, due to their high oxidation potentials, triggered by the electron-withdrawing effect of the substituted fluorines, and among the most popular ones stands tetrakis(pentafluorophenyl)porphyrin (2H-20F-TPP) due to its ease of synthesis and functionalization [11]. Heptazines, on the other hand, (Scheme 1) are a fascinating family of high nitrogen aromatic fused tricyclic heterocycles [12,13,14]. Because of their benzene-type aromaticity, they are fluorescent and due to their high nitrogen content, they own a high reduction potential, especially in the excited state because of their large bandgap. Their high electron deficiency actually situates them between triazines [15] and tetrazines [16], the two other common classes of analogous widely studied heterocycles. Indeed, due to their highly oxidizing excited state, heptazine can act as photocatalysts [17], and trigger irreversible photoinduced electron transfer to a nearby partner.

We reasoned that coupling an already electron-deficient porphyrin with an heptazine could photochemically enhance the oxidizing efficiency of the porphyrin, and possibly promote unusual oxidation states, in the case where a redox metal (e.g., iron) is present in the porphyrin cavity. Porphyrin dyads have been already described with fullerenes [18,19], cyclic paraquats [20], ferrocene [21] or TTF [22] partners, and have shown interesting photophysical properties. We present in this communication the first example of a dyad involving both a porphyrin and an heptazine ring, along with its electrochemical and spectroscopic characteristics.

## 2. Results and Discussion

**Figure 1:**
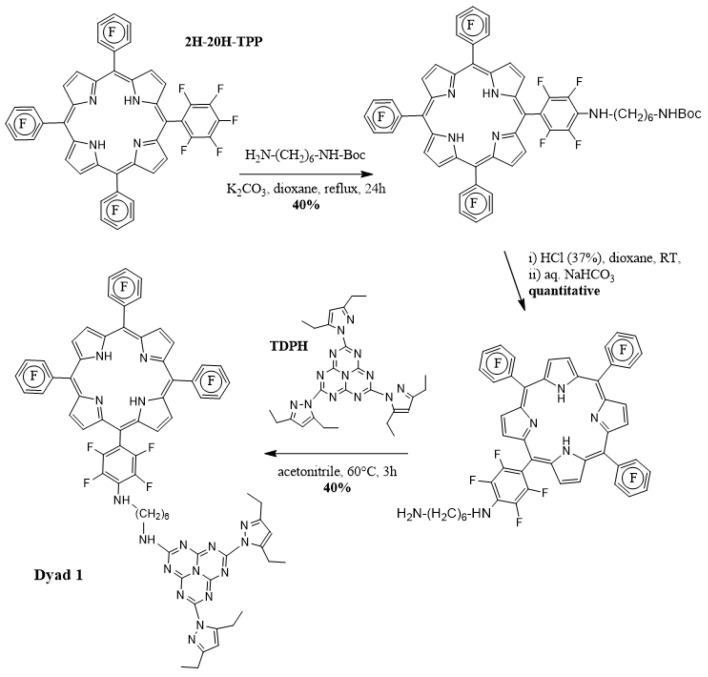
Synthetic Scheme of the porphyrin-heptazine dyad 1.

The new dyad and its synthetic scheme are presented on the following Figure 1.

**Figure 2:**
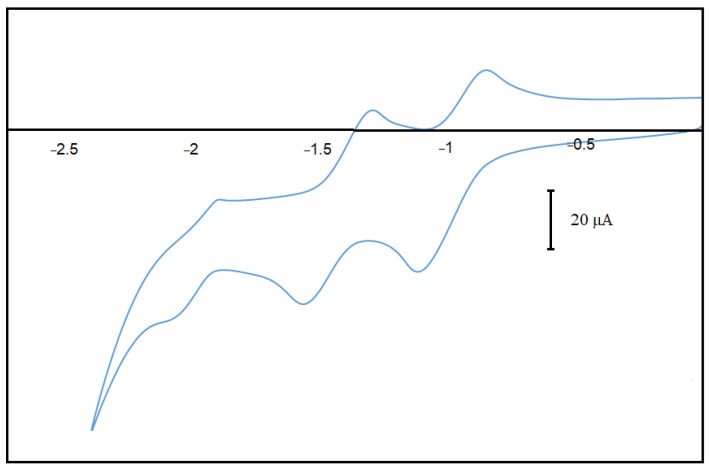
Reversible CVs of dyad 1 upon scanning in reduction to negative potentials.

The synthetic Scheme is straightforward, and benefits from the possibility to substitute with analogous success primary amines both on the perfluorinated porphyrin ring and the heptazine ring, using a mono-protected 1,6-hexane diamine. In a first step, one *p*-fluorine on the tetrakispentafluorophenylporphyrin was substituted with *N*-Boc-hexyl diamine by reacting the porphyrin with 0.6 equivalent of amine in presence of K_2_CO_3_ for 24 h at 100 °C. After chromatographic purification, the monosubstituted porphyrin was isolated in 40% yield. The Boc group was then removed by treatment with HCl (37%)/dioxane solution. Finally, the second aromatic substitution was realized by reacting one equivalent of TDPH with one equivalent of monosubstituted porphyrin in acetonitrile at 60 °C for 3 h. After chromatographic purification, the Dyad 1 was obtained in 40% yield. All spectroscopic structural characterizations (NMR, HRMS) have been made and are reported in the ESI section (Figure 2 and Figures S1–S9).

We have investigated the spectroscopic and electrochemical behavior of the dyad. It displays the porphyrins reduction at high potentials (due to the fluorine’s presence) followed by the partly reversible heptazine reduction at a lower potential (Figure 1).

Potential (a behavior very close to the analogous TDPH precursor) [23]. From the current intensities, it can be seen that the porphyrin exchanges two electrons successively, while the heptazine exchanges only one electron at lower potentials. Upon oxidation, an irreversible peak attributable to the porphyrin can also be observed (See ESI Figure S10). The CVs of the porphyrin precursors (amino-Boc and free amine) (see ESI Figures S11 and S12) show as expected exactly the same reduction potentials attributable to the porphyrin, but of course without the heptazine response.

**Figure 3:**
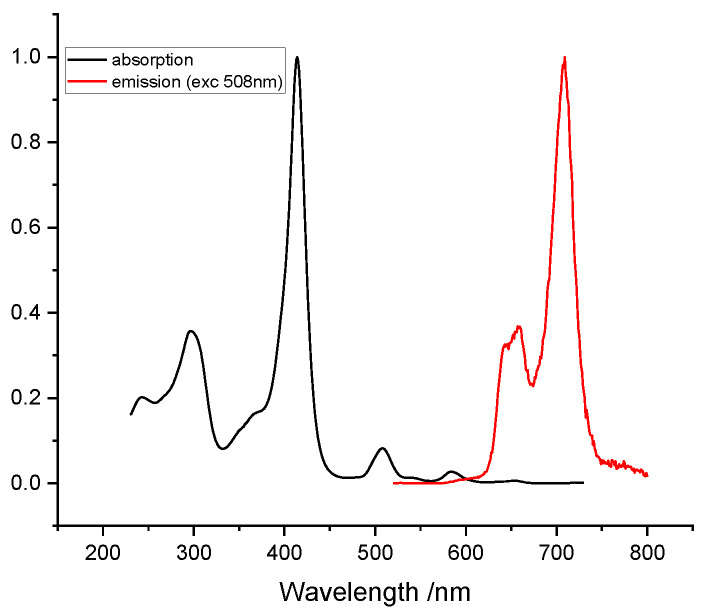
Absorption and fluorescence spectra of Dyad 1 in ethanol (See ESI for detailed exp. conditions).

**Figure 4:**
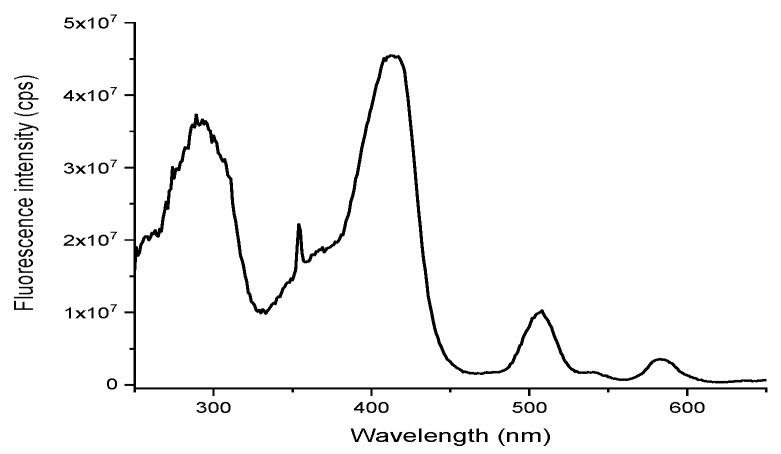
Excitation spectrum of dyad 1 in DCM at 710 nm.

The spectroscopic characteristics of our dyad are more surprising. The absorption and fluorescence spectra are displayed on Figure 3 above. The absorption spectrum displays nicely the heptazine ring absorption around 300 nm, followed by the intense Soret band of the porphyrin, blue shifted by the influence of fluorine, and the Q-bands afterwards. The excitation spectrum (Figure 4) is identical to the absorption spectrum, which indicates a standard photophysical behavior of the partners (e.g., excimer formation), along with a predominant energy transfer (in case of predominant electron transfer the porphyrin would be weakly emissive).

On the other hand, the emission spectrum displays almost only the porphyrin emission at 720 nm, but with a lower quantum yield (QY) of 0.03. Comparison with the generic tetrakis-pentafluorophenylporphyrin (2H-20F-TPP) shows an approximate 6% QY, while the emission wavelength remains practically the same (700 vs. 720 nm) [24,25]. These combined results show: (1) that the heptazine emission is totally quenched by transfer processes, and (2) that the decreased emission QY demonstrates that energy transfer is not the sole process since the very similar tris-(perfluorophenyl)-porphyrin alone displays a twofold QY decrease. It is likely to suppose that a concurrent electron transfer (SET) from the heptazine to the porphyrin occurs to some extent, and this is supported by the comparison of the oxidation potentials of the porphyrin and the heptazine’s excited state.

Actually, promotion of electron vs. energy transfer in Dyad 1 is reasonable, since the redox potential of 2H-20F-TPP has been measured of +1.52 V, very close to our measurements on Dyad 1, of approx. +1.51 V (+1.25 V vs. Ag/AgCl ref. check ESI Figure S10), the structural difference between the generic 2H-20F-TPP being minor. On the other hand, the redox potential in the excited state E_Hp_* of the substituted heptazine can be estimated (through Rehm-Veller’s approach) of E_Hp_* = −1.3 V + 3.85 V = + 2.55 V (1.3 V being the measured reduction potential for the heptazine ring, and 3.85 nm estimated value of the heptazine bandgap, taking the E_0-0_ at the onset of the spectra at 320 nm.) vs. SCE, which comes to about 2.8 V vs. NHE (Assuming a 0.24 V difference between the SCE and the NHE.). This high value explains probably the strong competition between electron vs. energy transfer, despite the fact that roughly half of the porphyrin ring fluorescence is retained. Another clue of the fact that electron transfer occurs, but to a limited extent, is the comparison of the emission spectra between two solvents of different polarities and dielectric constants, dioxane and DCM (ESI Figures S13 and S14). The remaining heptazine fluorescence is higher in DCM, as a probable consequence of a less efficient PET in this solvent.

For sake of comparison, we have in addition also prepared a molecule with exactly the isoelectronic heptazine core (featuring one primary amine and two pyrazoles) the 1,2-bis(*N*-diethylpyrazolyl)-3-[aminomethl-(1-adamandanyl)]-heptazine (DDPAH) (See Scheme S1 in ESI). We have chosen aminomethyladamantane as the amine, because of the easier preparation and purification induced by the bulky adamantine, while having no influence on the other hand on the electronic properties. This heptazine is electroactive (Figure S14) and fluorescent in the blue-violine domain, as it could be expected (see spectra in the ESI section, Figure S15). The fact that this heptazine with no porphyrin is intrinsically fluorescent (as it could be expected from our initially reported results on analogous compounds [12,23]) validates additionally the energy and charge transfer in the dyad. The electrochemical response in reduction of DDPAH is as expected around −1.75 V, at the same position than the last reduction peak of dyad 1.

## 3. Conclusions

We have been able to prepare for the first time a porphyrin-heptazine dyad and analyze its spectroscopic and electrochemical properties. This example is in addition the first example known to date of a bifunctional heptazine bearing photoactive and/or electroactive attached moieties. Our preliminary results indeed show that already oxidation electron transfer is promoted between the substituted heptazine excited state and the 2H-porphyrin. This should be enhanced in metalated porphyrins, the syntheses of which are planned in a near future.

## Data Availability

Not applicable.

## Supplementary Materials

The following supporting information can be downloaded at: https://www.mdpi.com/article/10.3390/molecules27196698/s1, Figure S1: DDPAH 1H spectrum (500 MHz, CDCl3); Figure S2: DDPAH 13C spectrum (125 MHz, CDCl3); Figure S3: 1H NMR Spectrum of TPPF19NH(CH2)6NHBoc (500 MHz, CDCl3); Figure S4: 1H NMR Spectrum of TPPF19NH(CH2)6NH2 (500 MHz, CDCl3); Figure S5: 19F NMR Spectrum of TPPF19NH(CH2)6NH2 (471 MHz, CDCl3); Figure S6: HRMS-ESI Spectrum of TPPF19NH(CH2)6NH2; Figure S7: 1H NMR Spectrum of Dyad 1 (500 MHz, CDCl3); Figure S8: 19F NMR Spectrum of Dyad 1 (471 MHz, CDCl3); Figure S9: HRMS-ESI Spectrum of Dyad 1; Figure S10: CV of dyad 1 over the whole potential range, including oxidation; Figure S11: CV in reduction of TPPF19NH(CH2)6NHBoc Ag/10-1M Ag+, checked against ferrocene); Figure S12: CV in reduction of TPPF19NH(CH2)6NH2 Ag/10-1M Ag+, checked against ferrocene); Figure S13: Fluorescence emission spectrum of dyad heptazine-porphyrin in DCM (black) and dioxane (red), upon 300nm excitation.; Figure S14: CV of aminomethyl(1-adamantanyl)-bis(diethylpyrazolyl)heptazine DDPAH; Figure S15: Absorption spectrum of DDPAH; Scheme S1: Scheme and Synthesis of DDPAH.
