# Supplementary material for: First Example of a Heptazine-Porphyrin Dyad; Synthesis and Spectroscopic Properties
Source: Molecules. 2022 Oct 8;27(19):6698. doi: 10.3390/molecules27196698 (PMC9571928; doi:10.3390/molecules27196698)
Supplement: Supplementary file 1 [file molecules-27-06698-s001.zip › molecules-1900551-supplementary.pdf]

<u>ESI for the article:</u> First example of a heptazine-porphyrine dyad; synthesis and spectroscopic properties.

General informations:

All chemicals products were purchased from commercial sources (Sigma-Aldrich or Fisher Scientific) and were used as received. Analytical thin layer chromatography (TLC, purchased from Merck, Darmstadt, Germany) was performed on Silica plates (Kieselgel60 F254, thickness 0.2 mm, Merck) and revealed by direct observation. Purifications were performed with Combiflash Rf 100® from Teledyne Isco. The stationary phase consisted of an 80 g silica column. The products to be purified were solubilized in a minimum amount of solvent and fixed on Florisil (60-100 mesh, VWR). For preparative thin layer chromatography, 20 X 20 cm glass plates were coated with an approximately 0.2 mm thin layer of Silica Gel (Merck Kieselgel silica gel 60 P). The plates were then dried at room temperature for 12 h, heated at 90°C for 2h (to remove any absorbed water), and cooled before use.

Spectroscopy

^{1}H and ^{19}F NMR spectra were recorded on a Bruker Avance III HD 500 MHz NMR spectrometer with $CDCl_3$ used as a solvent. MALDI-TOF spectra were performed on a Shimadzu AXIMA confidence using dithranol as matrix. High resolution electrospray ionization mass spectrometry (HR ESI-MS) was performed at the ICOA/CBM platform (Orléans University) on a Bruker Q-TOF maXis mass spectrometer, coupled to an Ultimate 3000 RSLC chain (Dionex). Absorption spectra were recorded on a Shimadzu UV-2501-PC spectrophotometer in $CHCl_3$ solutions. Fluorescence and excitation spectra were recorded using a SPEX Fluoromax-3 or on a Fluorolog-3 (Horiba Jobin-Yvon) spectrometer with CH_2Cl_2 (Spectro grade) used as a solvent.

Electrochemistry

Electrochemical studies were performed using acetonitrile (SDS, anhydrous for analysis) as a solvent, with N-tetrabutylammonium hexafluorophosphate (Fluka, puriss.) as the supporting electrolyte. The substrate concentration was ca. 0.5 mM. A 2 mm glassy carbon electrode was used as the working electrode, along with a Ag+/Ag (10^{-2} M) reference electrode and a Pt wire counter electrode. The cell was connected to a PAR Versastat 4 potentiostat monitored by a PC. The reference electrode was checked vs. ferrocene as recommended by IUPAC. All solutions were degassed by argon bubbling prior to each experiment.

5,10,15,20-tetrakis(pentafluorophenyl)porphyrin (**TPPF20**) was synthesized as described in the literature[1].

Synthesis of TPPF19NH(CH_2)$_6$NHBoc (2). A 100 mL round-bottom flask containing a magnetic stirring bar was charged with 5,10,15,20-tetrakis(pentafluorophenyl)porphyrin (549 mg, 0.56 mmol), potassium carbonate (66.5 mg, 0.42 mmol), *N*-Boc-1,6-diaminohexane (68 mg, 0.31 mmol) and dioxane (40 mL). The mixture was heated at 100 ◦C for 24 h. The flask was allowed to cool down to room temperature and the solvent was removed under reduced pressure. Then, the residue was diluted with chloroform and the organic phase was washed with water (3 × 50 mL). After separation of the organic phase, the solvent was removed under vacuum and the residue was purified by flash chromatography (gradient PE/AcOEt from 20% to pure AcOEt). From the first fraction was recovered the starting porphyrin (385.7 mg) and compound **2** was obtained from the second fraction in 40% yield (146.6 mg, 0.13 mmol).).

^1H NMR (500 MHz, $CDCl_3$): δ=9.03 (d, *J*=4.2 Hz, 2H, β-pyrrole CH), 8.89 (s, 4H, β-pyrrole C*H*), 8.88 (d, *J*=4.6 Hz, 2H, β-pyrrole C*H*), 4.58 (s, 1H, N*H*), 4.31 (s, 1H, N*H*), 3.70 (q, *J*=6.1 Hz, 2H, NHCH_2), 3.21 (q, *J*=6.1 Hz, 2H, CH_2NHCO), 1.85 (quint., *J*=7.3 Hz, 2H, $CH_2CH_2CH_2$), 1.60 (m, 4H, $CH_2CH_2CH_2$), 1.50 (m, 2H, $CH_2CH_2CH_2$), 1.48 (s, 9H, CCH_3), -2.89 ppm (br s, 2H, N*H*); ^{19}F NMR (471 MHz, $CDCl_3$) : δ = -185.13 to -184.87 (m, 6F, F-*meta*), -184.41 (d, J = 19.1 Hz, 2F, F'-*meta*), -175.17 to -174.94 (m, 3F, F-*para*), -164.22 (d, J = 19.1 Hz, 2F, F'-*ortho*), -159.98 (d, J = 22.5 Hz, 6F, F-*ortho*); MS (MALDITOF): m/z: calcd for $C_{55}H_{33}F_{19}N_6O_2$: 1170.21; found: 1171.15 $[M+H]^+$.

Synthesis of TPPF19NH(CH_2)$_6$$NH_2$ (3). In a round-bottom flask containing porphyrin **2** (347.9 mg) and dioxane (15 mL), it was added HCl, 37% (15 mL). The resulting mixture was maintained under stirring for 2 h at room temperature (until starting material was consumed -TLC monitoring). Then, the reaction was neutralized with a saturated bicarbonate solution. Finally, the organic product was extracted with chloroform, the organic phase separated, dried over anhydrous $MgSO_4$ and the solvent removed under reduced pressure. The product **3** was obtained in quantitative yield.

^1H NMR (500 MHz, $CDCl_3$): δ=9.01 (br s, 2H, β-pyrrole CH), 8.88 (m, 6H, β-pyrrole C*H*), 4.26 (br s, 1H, N*H*), 3.56 (br s, 2H, NHCH_2), 2.95 (br s, 2H, C$H_2$$NH_2$), 2.38 (br s, 2H, N$H_2$), 1.72 (br s, 4H, $CH_2CH_2CH_2$), 1.47 (br s, 4H, $CH_2CH_2CH_2$), -2.90 ppm (br s, 2H, N*H*); ^{19}F NMR (471 MHz, $CDCl_3$) : δ = -185.16 to - 184.83 (m, 6F, F-*meta*), -184.44 (d, J = 19.0 Hz, 2F, F'-*meta*), -175.16 to -174.85 (m, 3F, F-*para*), -164.25 (d, J = 19.3 Hz, 2F, F'-*ortho*), -160.12-159.88 (m, 6F, F-*ortho*); HRMS (ESI): m/z = $C_{50}H_{26}F_{19}N_6$, $[M+H]^+$, 1071.191007, calcd. 1071.190715 found.

Synthesis of Dyad 1. 2,5,8-tris(3,5-diethyl-pyrazolyl)-heptazine (159 mg, 0.29 mmol) and 5-(4-{[6-aminohexyl]amino}-2,3,5,6-tetrafluorophenyl)-10,15,20-tris(pentafluorophenyl)porphyrin (316,2 mg, 0,30 mmol) were dissolved in 14 mL acetonitrile. The mixture was heated at 60°C for 3 hours. The solvent was then evaporated and the crude product was purified by preparative thin-layer chromatography plate (Mobile phase: AcOEt). The dyad 1 was obtained in 41% yield (180.1 mg, 0.12 mmol).

^1H NMR (500 MHz, $CDCl_3$): δ= 9.03 (d, *J*=4.3 Hz, 2H, β-pyrrole CH), 8.89 (s, 4H, β-pyrrole C*H*), 8.87 (d, *J*=4.6 Hz, 2H, β-pyrrole C*H*), 6.25 (t, *J*=5.3 Hz, 1H, N*H*), 6.165 (s, 1H, pyrazolyl C*H*), 6.160 (s, 1H, pyrazolyl C*H*), 4.31 (br s, 1H, N*H*), 3.71 (q, *J*=6.7 Hz, 2H, NHCH_2), 3.66 (q, *J*=6.9 Hz, 2H, CH_2NHCO), 3.26 (q, *J*=7.3 Hz, 2H, pyrazolyl CH_2), 3.19 (q, *J*=7.3 Hz, 2H, pyrazolyl CH_2), 2.73 (q, *J*=7.6 Hz, 2H, pyrazolyl CH_2), 2.71 (q, *J*=7.6 Hz, 2H, pyrazolyl CH_2), 1.85 (quint., *J*=7.2 Hz, 2H, $CH_2CH_2CH_2$), 1.73 (quint., *J*=7.4 Hz, 2H, $CH_2CH_2CH_2$), 1.60 (quint., *J*=7.5 Hz, 2H, $CH_2CH_2CH_2$), 1.53 (quint., *J*=7.5 Hz, 2H, $CH_2CH_2CH_2$), 1.294 (t, *J*=7.4 Hz, 3H, pyrazolyl CH_3), 1.292 (t, *J*=7.4 Hz, 3H, pyrazolyl CH_3), 1.26 (t, *J*=7.2 Hz, 3H, pyrazolyl CH_3), 1.25 (t, *J*=7.2 Hz, 3H, pyrazolyl CH_3), -2.89 ppm (br s, 2H, N*H*); ^{19}F NMR (471 MHz, $CDCl_3$) : δ = -185.10 to - 184.86 (m, 6F, F-*meta*), -184.33 (d, J = 18.9 Hz, 2F, F'-*meta*), -175.12 to -174.91 (m, 3F, F-*para*), -164.13 (d, J = 19.1 Hz, 2F, F'-*ortho*), -159.9 (d, J = 22.2 Hz, 6F, F-*ortho*). HRMS (ESI): m/z = $C_{70}H_{47}F_{19}N_{17}$, $[M+H]^+$, 1486.389147 calcd. 1486.368678 found.

Scheme and Synthesis of DDPAH

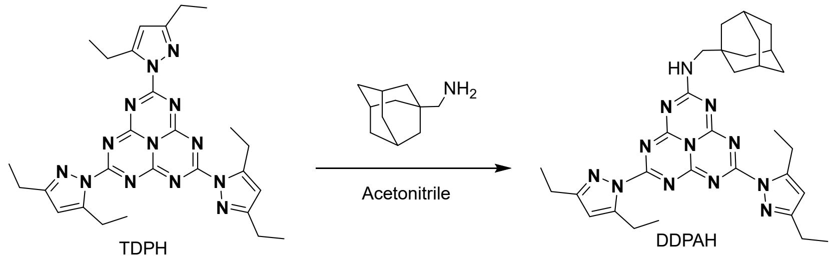

DDPAH was simply obtained by reacting 0.5 g TDPH at RT in 20 ml of acetonitrile with 0.9 eq of 1-(aminomethyl)-adamantane (Aldrich), followed by LPLC flash chromatography (gradient 0.5EP/0.5EtOAc to pure EtOAc), Yield 30% of a white powder. A slightly impure faction containing traces of TDPH was also collected.

^{1}H NMR (500 MHz, $CDCl_3$): δ= 1.25 (t., *J*=7.5 Hz, 2H, two pyrazolyl CH_2**CH_3**), 1.276 (m, adamantane C*H_2*), 1.530 (d, *J*=3.0 Hz, 6H, adamantane C*H_2*, next to aminomethyl groups), 2.648 (q, *J*=8 Hz, 2H, pyrazolyl **CH_2**-C*H_3*), 3.15-3.25 (m, 2H, pyrazolyl **CH_2**-C*H_3*) 5.9 ppm (s, pyrazole), 6.16 ppm, d, NH-**CH_2**-Ad), 6.37 ppm, t, **NH**-CH_2-Ad).

^{13}C NMR (125 MHz, $CDCl_3$): δ= Five aliphatic in the 10-30 region: 3 adamantanyl and the CH_3's of ethylpyrazoles. 34.2, C1 (substituted) of adamantanyl rest). 37.0, 40.14, CH_2's of ethylpyrazoles. 53.0, CH_2-NH. 101.22 CH pyrazole ring, 109.4, 109.5, heptazine connected to NH and para, 150.2, 150.5 pyrazoles, 157.0, 160.2 remaining heptazine Cs. The fact that all carbons can be seen individually stems from the fact that the heptazine is not symmetrical, the hindered adamantane moiety being locked on one side of the ring.

NMR and HRMS Spectra

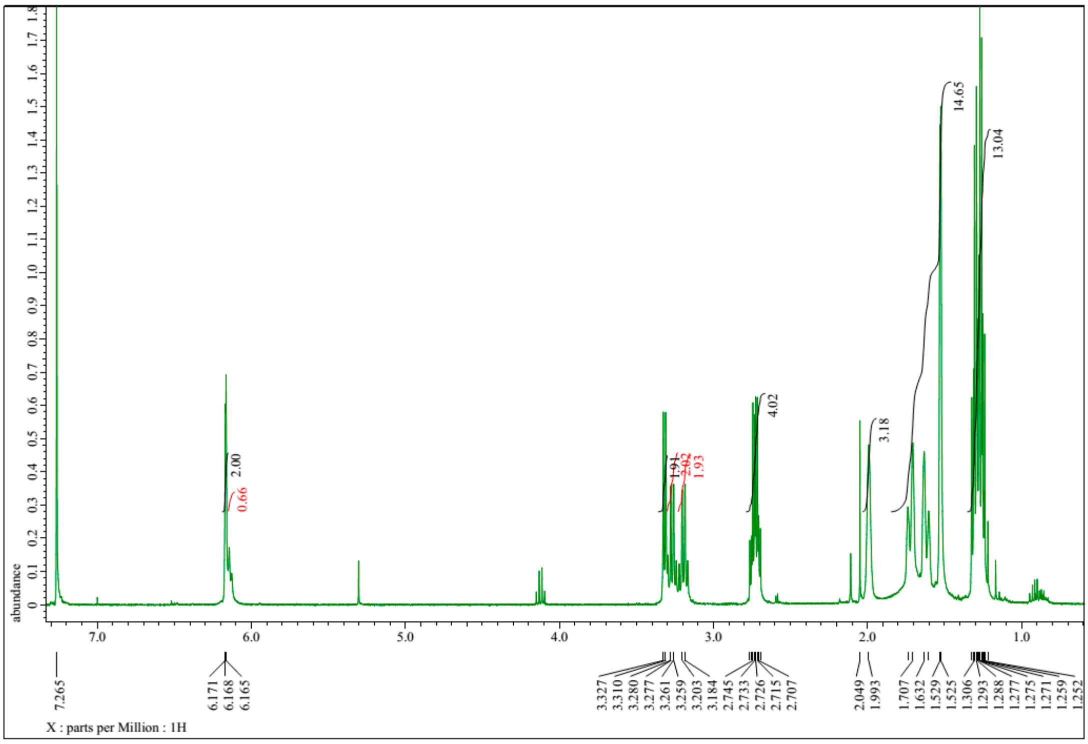

ESI Fig. S1 DDPAH ^{1}H spectrum (500 MHz, $CDCl_3$)

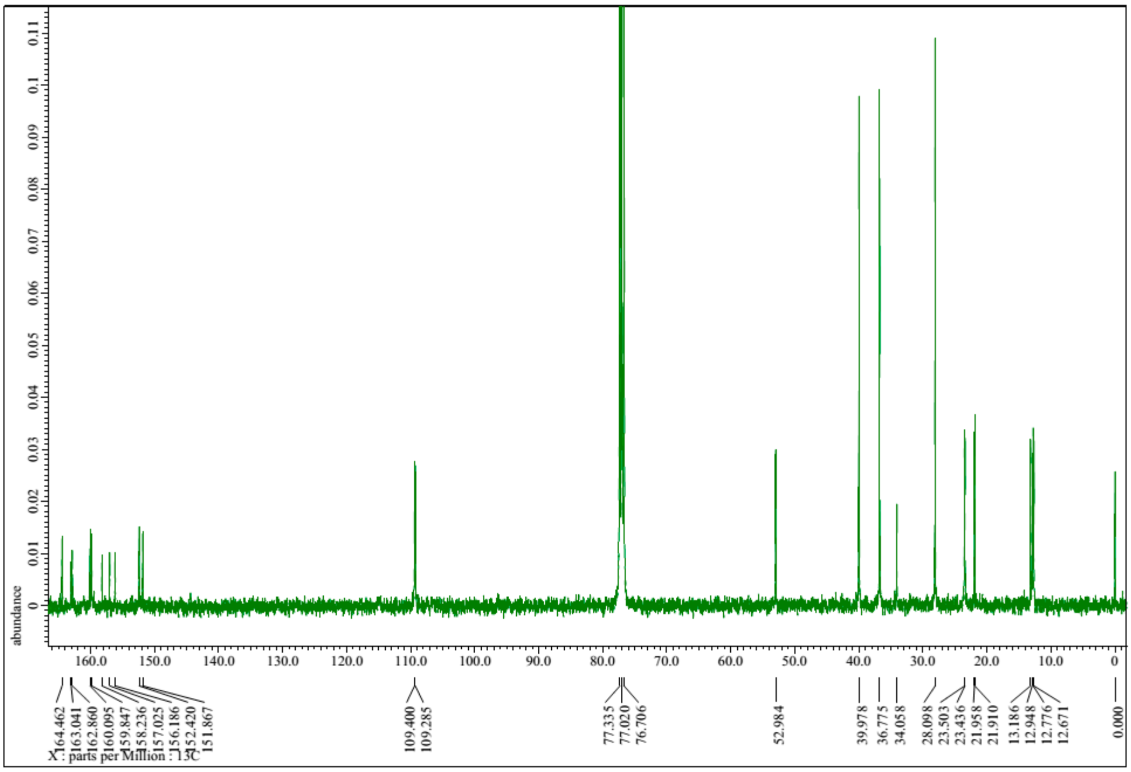

ESI Fig. S2 DDPAH ^{13}C spectrum (125 MHz, $CDCl_3$).

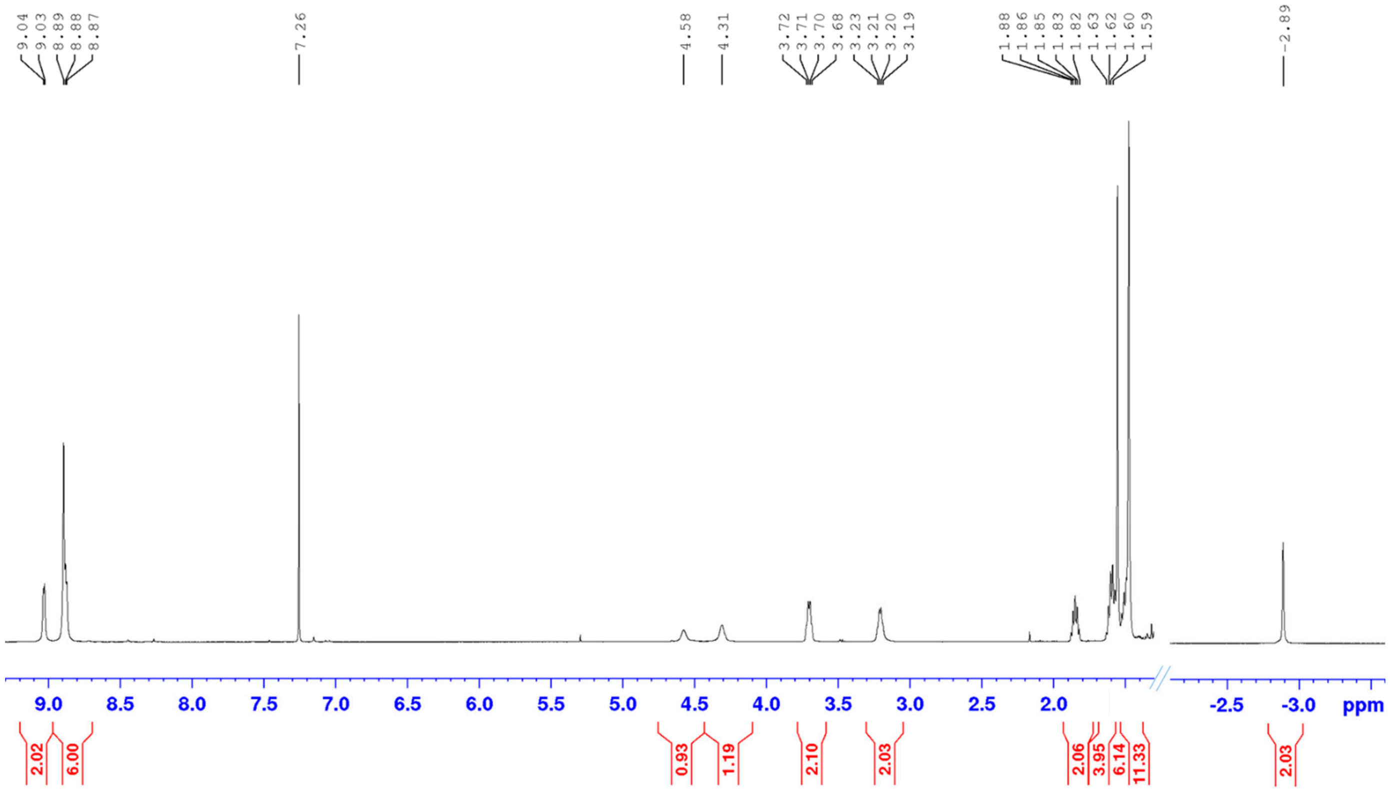

Figure S 3 . ^{1}H NMR Spectrum of ***TPPF19NH(CH$_2$)$_6$NHBoc*** *(500 MHz, CDCl$_3$)*

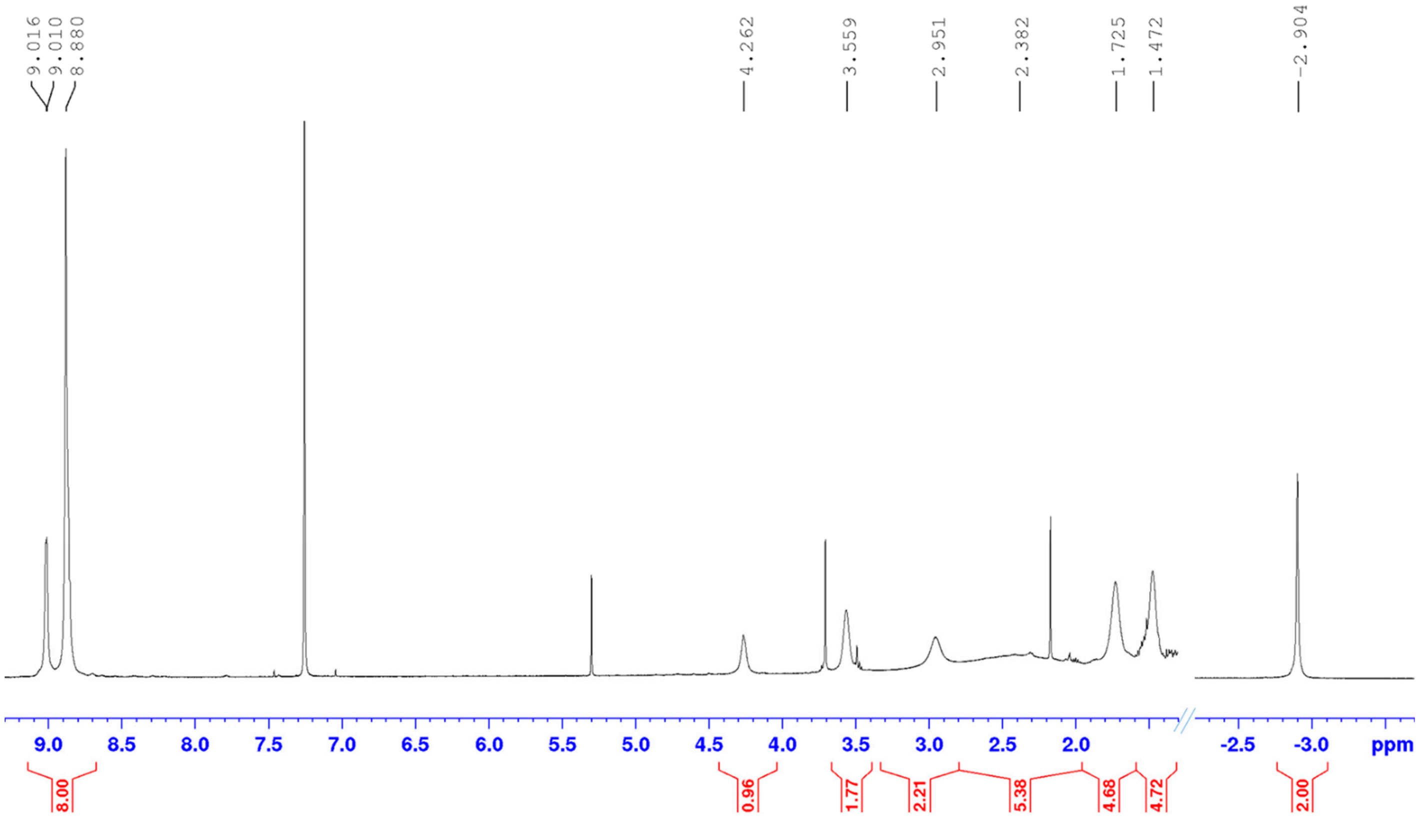

*Figure S 4 . ^{1}H NMR Spectrum of **TPPF19NH(CH$_2$)$_6$NH$_2$** (500 MHz, CDCl$_3$)*

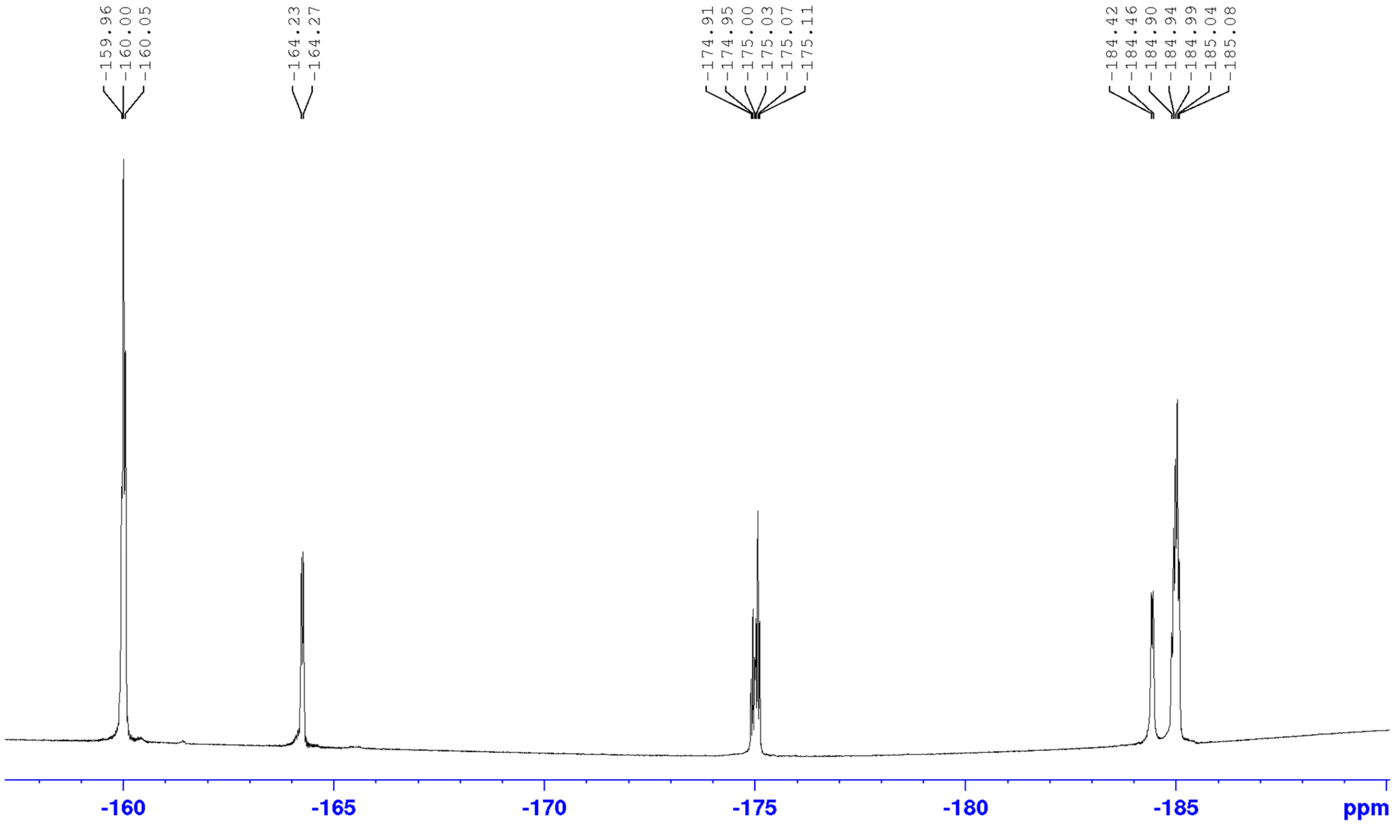

Figure S 5. ^{19}F NMR Spectrum of ***TPPF19NH(CH$_2$)$_6$NH$_2$*** (471 MHz, $CDCl_3$)

Acquisition Parameter

Source Type	ESI	Ion Polarity	Positive	Set Nebulizer	0.6 Bar
Scan Begin	50 m/z	Set Capillary	4500 V	Set Dry Heater	200 °C
Scan End	3000 m/z	Set Collision Cell RF	1800.0 Vpp	Set Dry Gas	7.0 l/min

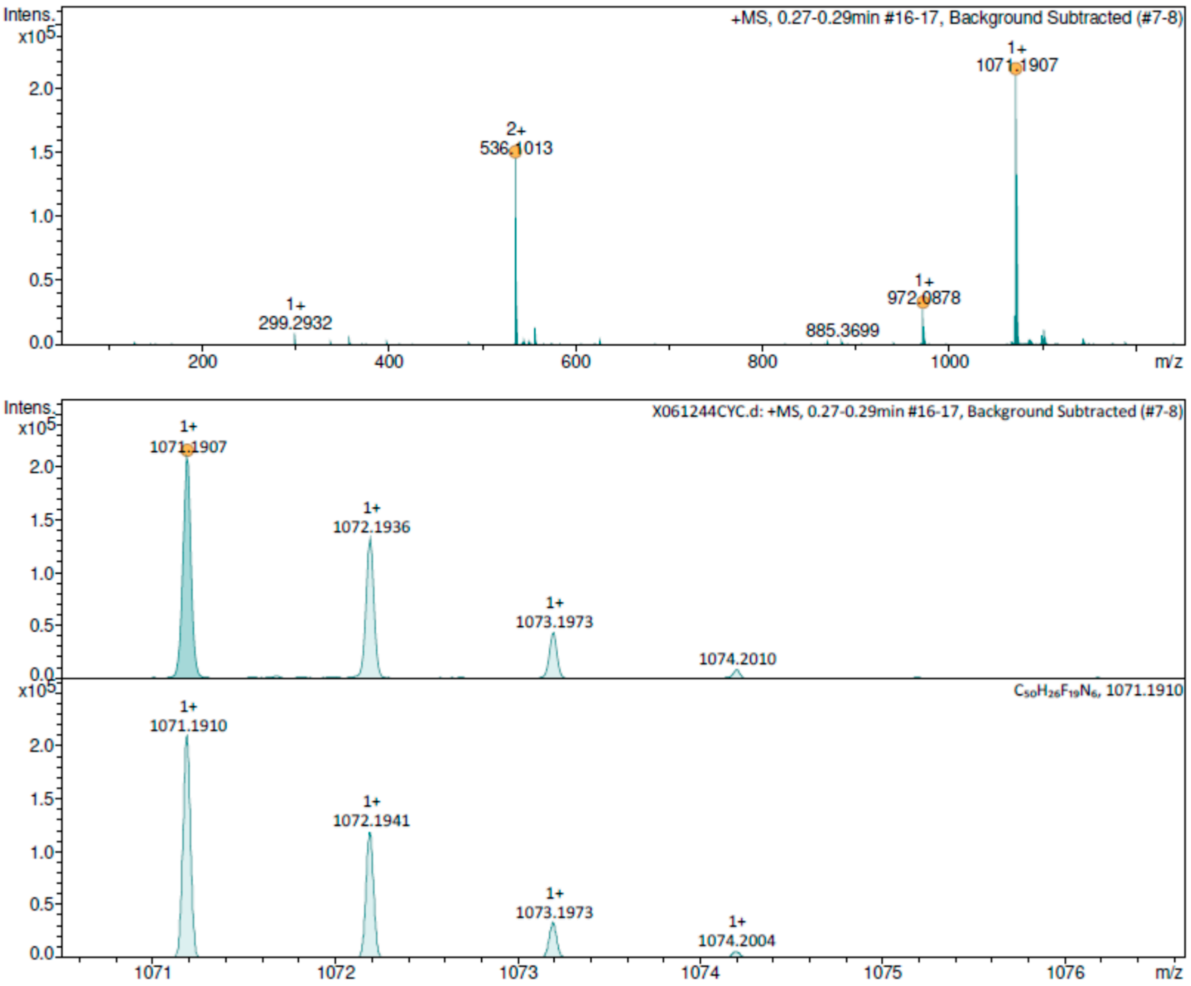

Figure S 6. HRMS-ESI Spectrum of ***TPPF19NH(CH$_2$)$_6$NH$_2$***

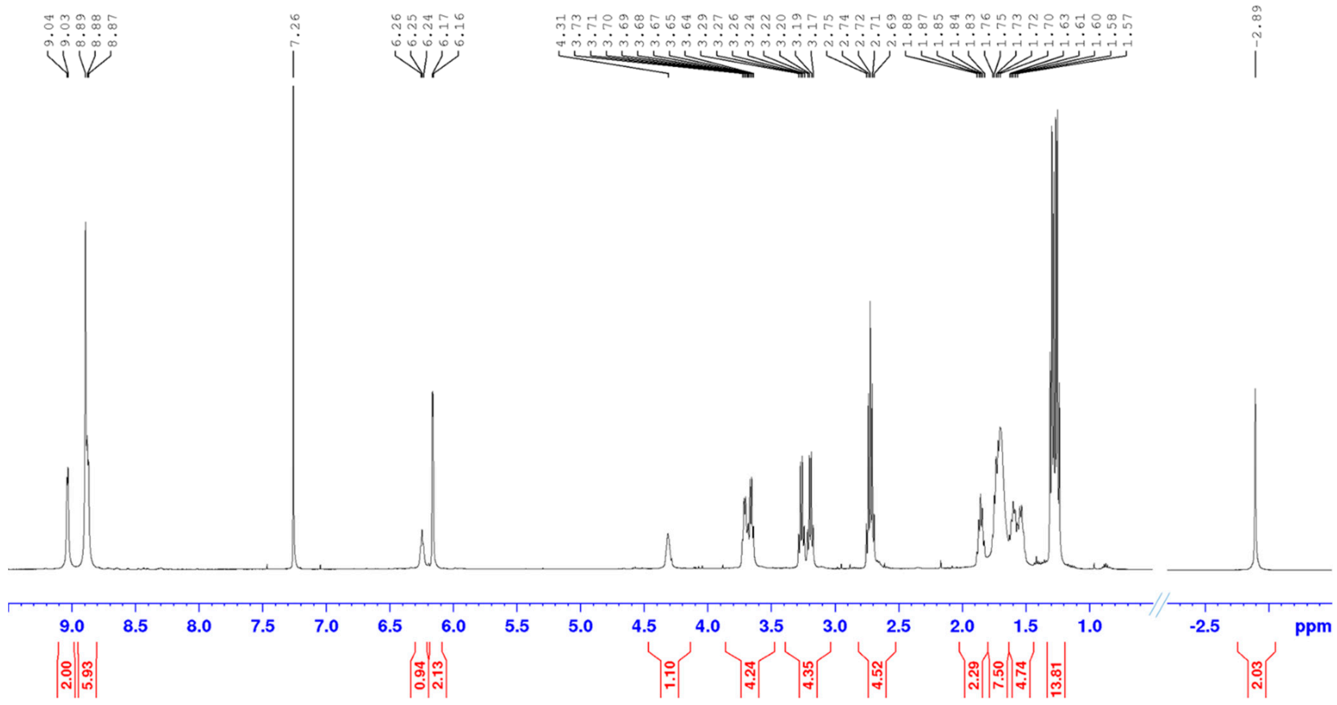

*Figure S 7. ^1H NMR Spectrum of **Dyad 1*** (500 MHz, $CDCl_3$)

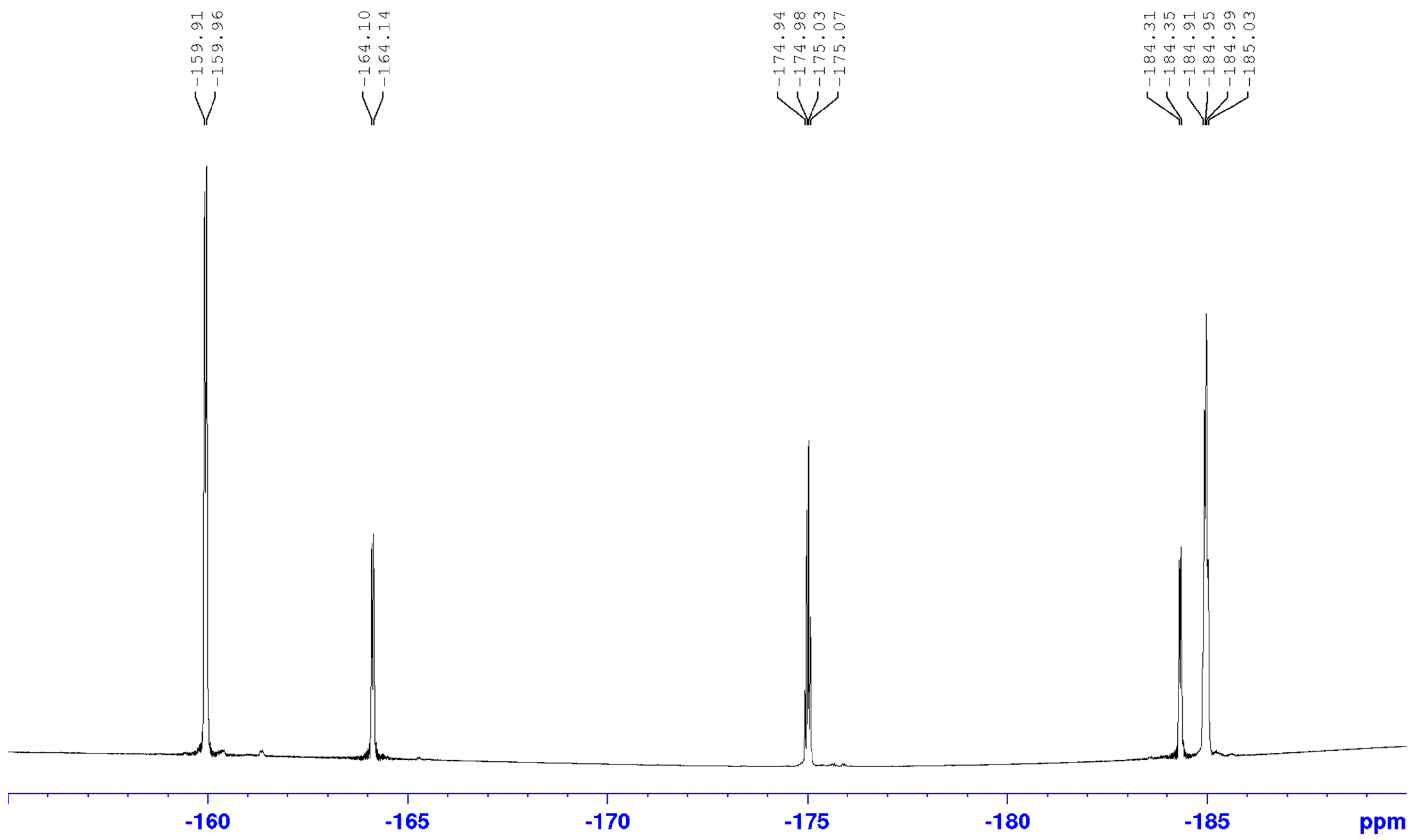

*Figure S 8. ^{19}F NMR Spectrum of **Dyad 1*** (471 MHz, $CDCl_3$)

Acquisition Parameter

Source Type	ESI	Ion Polarity	Positive	Set Nebulizer	0.6 Bar
Scan Begin	50 m/z	Set Capillary	4500 V	Set Dry Heater	200 °C
Scan End	3000 m/z	Set Collision Cell RF	1800.0 Vpp	Set Dry Gas	7.0 l/min

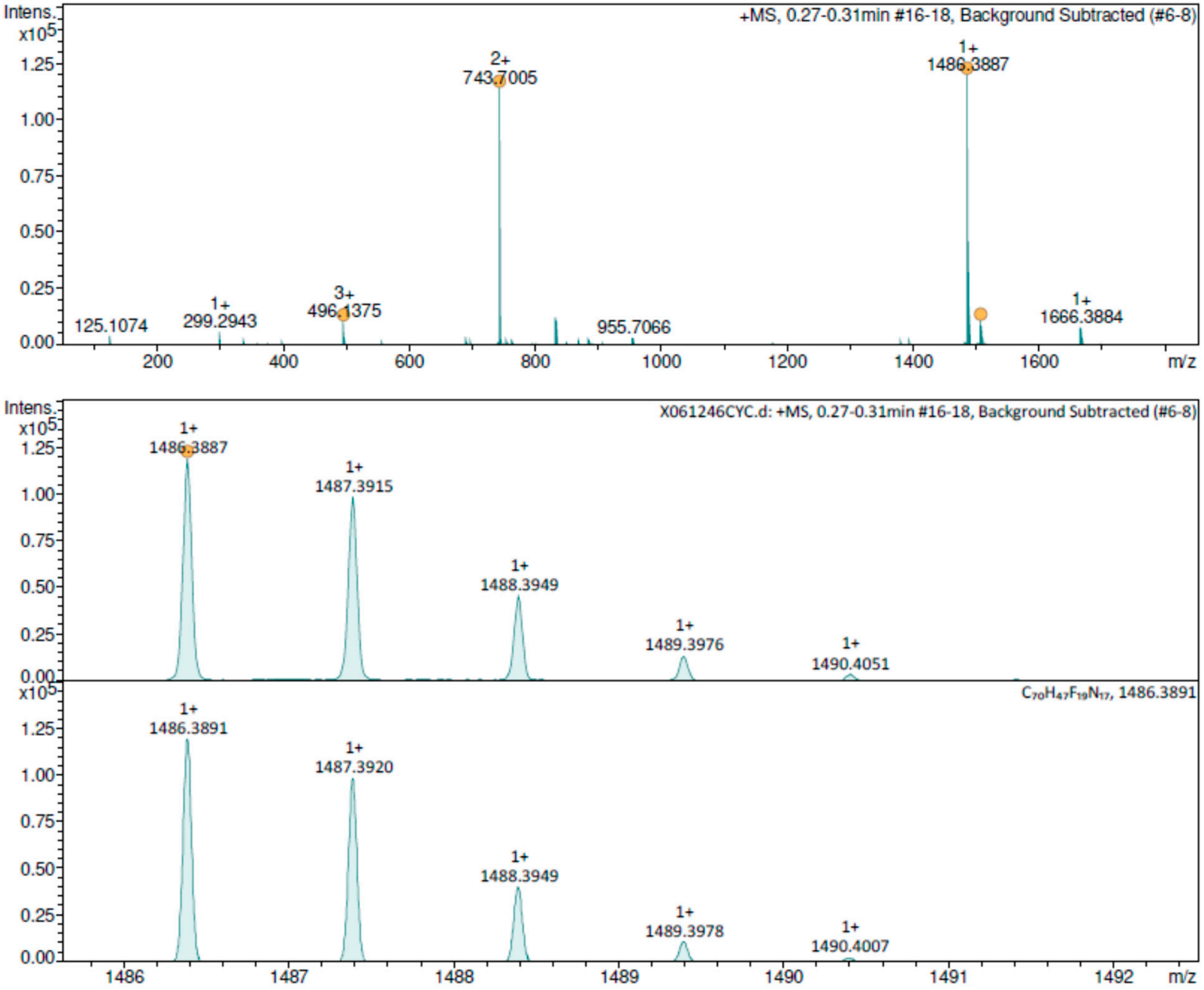

*Figure S 9. HRMS-ESI Spectrum of **Dyad 1***

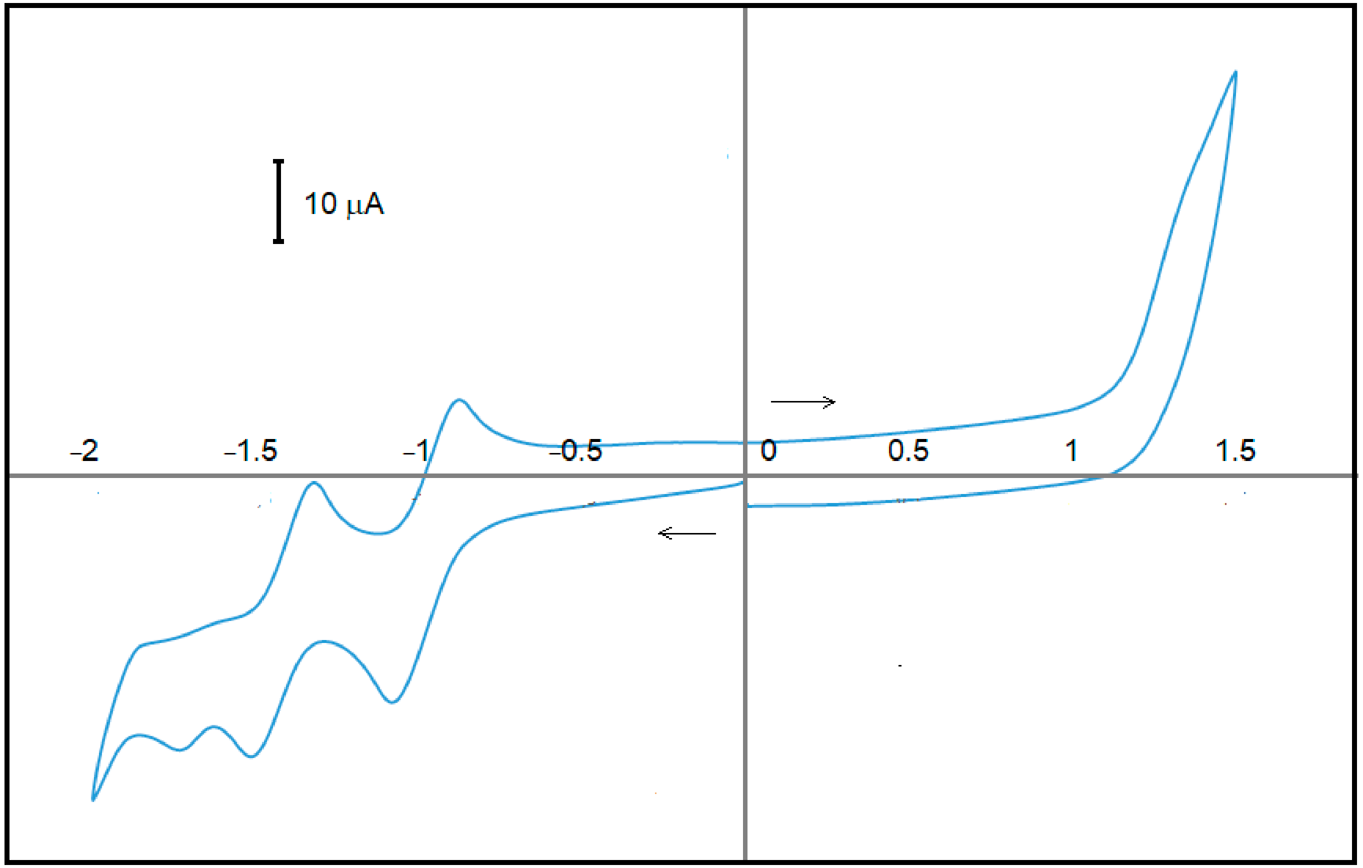

ESI Figure S10: CV of dyad 1 over the whole potential range, including oxidation

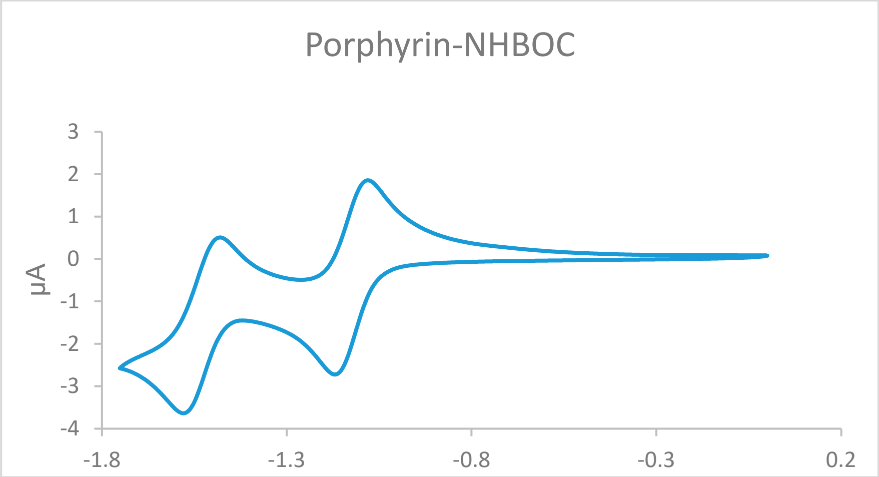

ESI Figure S 11: CV in reduction of **TPPF19NH(CH_2)$_6$NHBoc** *Ag/10^{-1}M Ag^+, checked against ferrocene)*

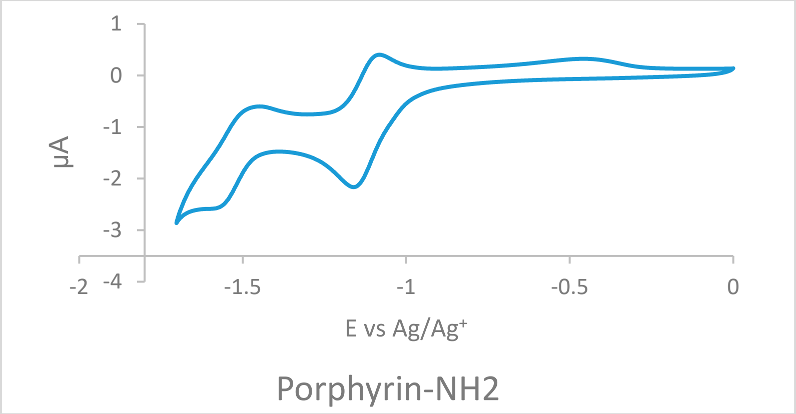

ESI Figure S 12: CV in reduction of **TPPF19NH(CH_2)$_6$NH2** *Ag/10^{-1}M Ag^+, checked against ferrocene)*

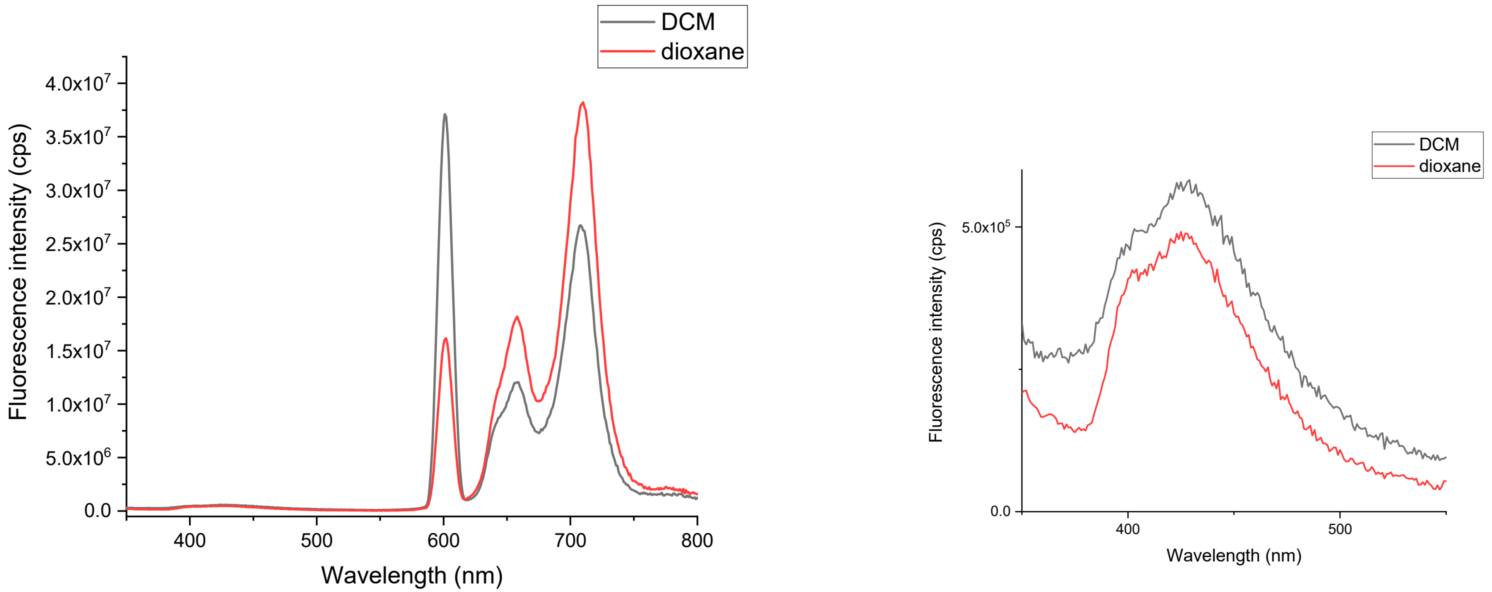

Figure S13 Fluorescence emission spectrum of dyad heptazine-porphyrin in DCM (black) and dioxane (red), upon 300nm excitation. Both solutions have similar absorbance (OD=0.11 at 414nm for dioxane solution and OD=0.10 at 414nm for DCM solution). Peak at 600nm is an artefact corresponding to twice the excitation wavelength. Left is the full emission spectrum, right is a zoom on the region corresponding to theweak remaining fluorescence of the heptazine.

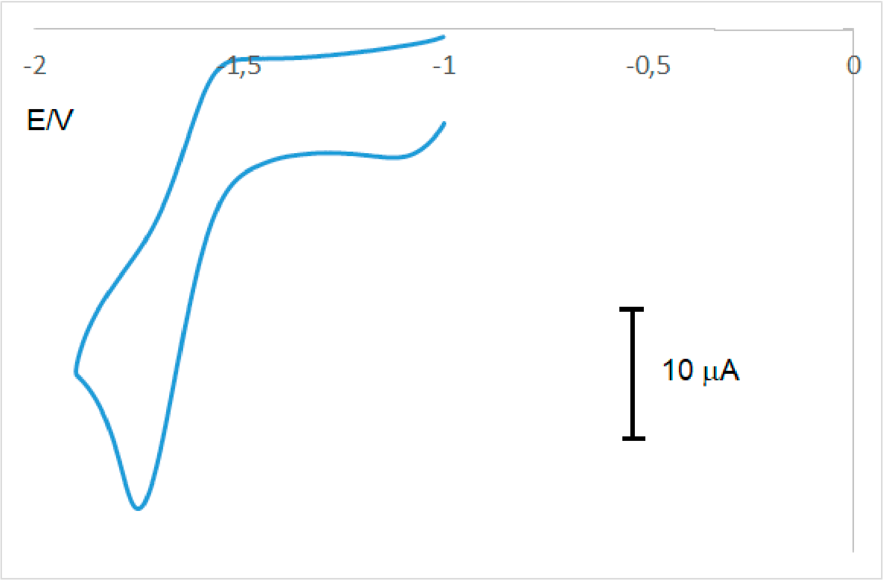

ESI Figure S 14: CV of aminomethyl(1-adamantanyl)-bis(diethylpyrazolyl)heptazine DDPAH.

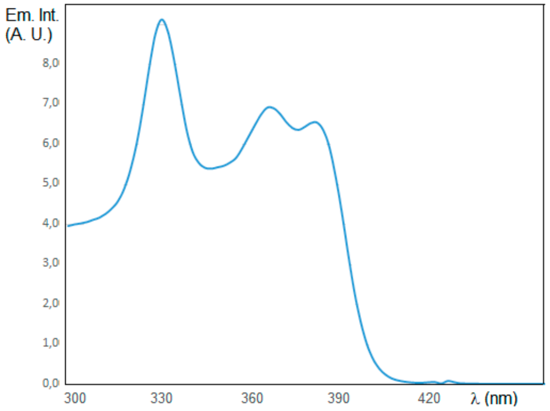

ESI Figure S15 Absorption spectrum of DDPAH

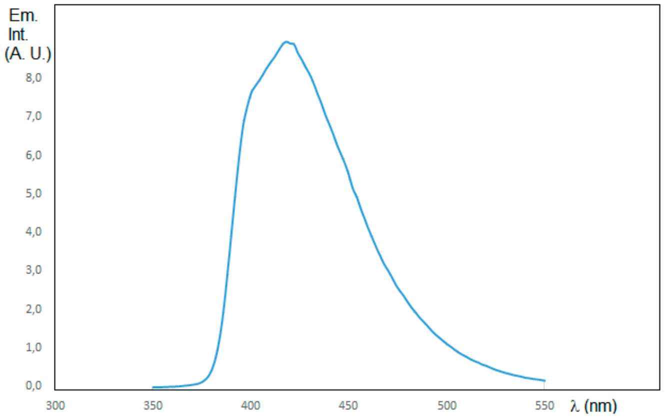

Figure S15 (bis) Fluorescence) spectrum of DDPAH
